# Microwave Ablation Combined With Chemotherapy Versus Chemotherapy Alone in Patients With Advanced Non‐Small Cell Lung Cancer—Systematic Review and Meta‐Analysis

**DOI:** 10.1111/1759-7714.70221

**Published:** 2026-01-09

**Authors:** Paul C. Onyeji, Amrinder Kaur, Leo Consoli, Shivank Dani, Sonise Momplaisir‐Onyeji, Felipe S. Passos, Torsten Doenst, Hristo Kirov, Tulio Caldonazo

**Affiliations:** ^1^ All Saints University School of Medicine Roseau Commonwealth of Dominica; ^2^ University of Chicago Chicago Illinois USA; ^3^ Federal University of Bahia Salvador Brazil; ^4^ GMERS, Medical College and Hospital, Sola Ahmedabad India; ^5^ School of Medicine Faculty of Medicine American University of Barbados Bridgetown Barbados; ^6^ Department of Thoracic Surgery MaterDei Hospital Salvador Brazil; ^7^ Department of Cardiothoracic Surgery Jena University Hospital, Friedrich‐Schiller‐University Jena Jena Germany

**Keywords:** chemotherapy, microwave ablation, non‐small cell lung cancer

## Abstract

Non‐small cell lung cancer (NSCLC) remains the leading cause of cancer‐related mortality worldwide. Although chemotherapy remains a cornerstone in the treatment of advanced NSCLC, local ablative strategies such as microwave ablation (MWA) have emerged as promising adjunctive therapies. However, the survival benefits of combining MWA with chemotherapy remain uncertain. This meta‐analysis aims to evaluate the efficacy and safety of microwave ablation combined with chemotherapy compared to chemotherapy alone in patients with advanced NSCLC. A comprehensive search was conducted in MEDLINE, EMBASE, and Cochrane Library through January 2025. Eligible studies included randomized controlled trials or observational studies comparing MWA associated with chemotherapy versus chemotherapy alone in patients with advanced NSCLC. The primary outcome was progression‐free survival (PFS); secondary outcomes included partial remission (PR) rate and adverse events (AE). Hazard ratios (HR) and risk ratios (RR) with 95% confidence intervals (CI) were pooled using random‐effects models. Individual patient data (IPD) were reconstructed from Kaplan–Meier curves to perform a one‐stage survival meta‐analysis. Four studies comprising 483 patients were included. MWA associated with chemotherapy significantly improved PFS (HR 0.408; 95% CI 0.24–0.49; *p* < 0.001; *I* ^2^ = 53.3%). No significant differences were found for PR (RR 0.74; 95% CI 0.37–1.50; *p* = 0.41) or AE (RR 1.08; 95% CI 0.86–1.36; *p* = 0.49). Sensitivity analyses confirmed the robustness of the findings. MWA combined with chemotherapy significantly improves PFS in advanced NSCLC without increasing toxicity.

## Graphical abstract

Microwave Ablation Plus Chemotherapy versus Chemotherapy Alone in Advanced NSCLC.

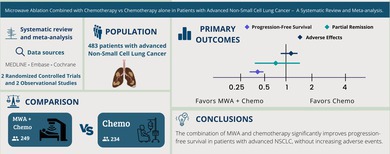

## 1 Introduction

Cancer remains one of the leading global health burdens, with the Global Cancer Observatory (GCO) 2020 identifying lung cancer as the second most diagnosed malignancy worldwide, following breast cancer. Despite this, it remains the leading cause of cancer‐related mortality, underscoring its disproportionate impact on public health [1]. In the United States, over 57% of lung cancer cases are diagnosed at an advanced stage, with distant metastases already present at the time of diagnosis [2]. Among the various types of lung cancer, non‐small cell lung cancer (NSCLC) accounts for approximately 85% of all lung cancer cases [3], highlighting the urgent need for effective strategies for effective prevention, early detection and management of this highly prevalent and lethal disease.

The therapeutic landscape for NSCLC is complex and stage dependent. While surgical resection offers the best chance for cure in patients with early‐stage disease, those diagnosed at stages II to IV typically require multimodal approaches, including chemotherapy, radiotherapy, and more recently, immunotherapy or targeted therapies tailored to specific genetic mutations [4, 5, 6]. For patients who are not surgical candidates or who have limited response to systemic therapies, local ablative techniques—such as radiofrequency ablation (RFA), cryoablation, and microwave ablation (MWA)—have emerged as minimally invasive alternatives. MWA employs non‐ionizing electromagnetic waves to generate localized heat and induce tumor necrosis through cellular disruption [7]. It has become an established tool in the treatment of various solid tumors, including those of the liver, lungs, kidneys, adrenal glands, and bone metastases [8].

Although MWA is a promising modality for local tumor control, its combined use with chemotherapy in advanced NSCLC remains insufficiently studied. Preliminary data suggest that this combination may enhance treatment efficacy by leveraging the local cytoreductive effect of ablation with the systemic reach of chemotherapy. This systematic review and meta‐analysis aims to evaluate clinical outcomes related to the effect of MWA combined with chemotherapy versus chemotherapy alone in patients with advanced NSCLC.

## 2 Methods

The study selection followed the Preferred Reporting Items for Systematic Reviews and Meta‐Analysis (PRISMA) statement guidelines [9, 10]. The review was registered in the International Prospective Register of Systematic Reviews (PROSPERO ID CRD420250644356).

### 2.1 Search Strategy

A comprehensive literature search was performed on MEDLINE, EMBASE, and the Cochrane Library from inception to June 2025 with the following search terms: “non‐small cell lung cancer,” “lung adenocarcinoma,” “microwave ablation,” and “chemotherapy.” We also searched for additional studies using the references of previously included studies. The complete search strategy is available in Table S1.

### 2.2 Study Selection

Two independent reviewers (P.C.O. and A.K.) screened the records, after de‐duplication. Any discrepancies and disagreements were resolved by a third author (F.S.P.). Titles and abstracts were reviewed against predefined inclusion and exclusion criteria.

### 2.3 Eligibility Criteria

Inclusion in this meta‐analysis was restricted to studies that met all the following eligibility criteria: (1) randomized controlled trials (RCTs) or observational studies; (2) published in English; (3) comparing MWA combined with chemotherapy versus chemotherapy alone in patients with advanced NSCLC (stage III–IV); and (5) reporting at least one outcome of interest. Exclusion criteria included studies involving animal models, case reports, conference abstracts; and non‐comparative study designs. Detailed information on chemotherapy regimens was inconsistently reported across the included studies and could not be reliably extracted, precluding comparative or subgroup analyses based on specific chemotherapy protocols.

### 2.4 Quality Assessment and Publication Bias

The quality of included studies was assessed using the Cochrane Collaboration tool for assessing the risk of bias in non‐randomized studies (ROBINS‐I) tool for observational studies [11] and the Risk of Bias 2 (RoB 2) tool for RCTs [12] (Figure S1). Publication bias was assessed for the primary outcome.

### 2.5 Data Extraction and Baseline Characteristics

Two authors (P.C.O. and S.M.) independently performed data extraction. Disagreements were resolved by consensus. The extracted variables included study characteristics (author, year of publication, country, sample size, and reported outcomes) and well as patient demographics (age, sex, Eastern Cooperative Oncology Group (ECOG) performance status, type of cancer, and tumor size).

### 2.6 Outcomes

The primary outcome was Progression‐free Survival (PFS). Secondary outcomes included partial remission rate and adverse events rate.

### 2.7 Statistical Analysis

Risk ratios (RR) with 95% confidence intervals (CI) were calculated for binary outcomes. A time‐to‐event data strategy was utilized for progression‐free survival. Cochran Q test and *I* ^2^statistics were used to assess heterogeneity; P values inferior to 0.10 and *I* ^2^ > 25% were considered significant for heterogeneity. A *p*‐value < 0.05 was considered for significant difference between the two groups. DerSimonian–Laird random effects models were used for all endpoints. The Cochrane Handbook for Systematic Reviews of Interventions was used for data handling and conversion. To ensure robustness of our findings, a two‐stage meta‐analysis and leave‐one‐out sensitivity analysis were also performed. All statistical analyses were performed using R (version 4.4.0, R Foundation for Statistical Computing, Vienna, Austria) and STATA IC17.0 (StataCorp LLC, College Station, Texas).

### 2.8 Individual Patient Survival Data Meta‐Analysis

We used the methods described by Wei et al. [13] to reconstruct individual patient data (IPD) from the Kaplan–Meier curves of all eligible studies for PFS. Raster and Vector images of the Kaplan–Meier survival curves were pre‐processed and digitized, so that the values reflecting to specific timepoints with their corresponding PFS information could be extracted. Where additional information (e.g., number‐at‐risk tables or total number of events) were available, they were used to further calibrate the accuracy of the time‐to‐event data. To confirm the quality of the timing of failure events captured, we thoroughly checked the consistency with the reported data provided in the original publications.

### 2.9 Meta‐Analysis of Reconstructed Data—One‐Stage Survival Meta‐Analysis

The Kaplan–Meier method [14] was used to calculate the PFS rate. The Cox proportional hazards regression model was used to assess between‐group differences. For these Cox models, the proportional hazards assumption was verified by plotting scaled Schoenfeld residuals, and log–log survival plots. We plotted PFS curves using the Kaplan–Meier product limit method and calculated the Hazard Ratios (HRs) and 95% CIs of each group.

## 3 Results

### 3.1 Study Characteristics

**FIGURE 1:**
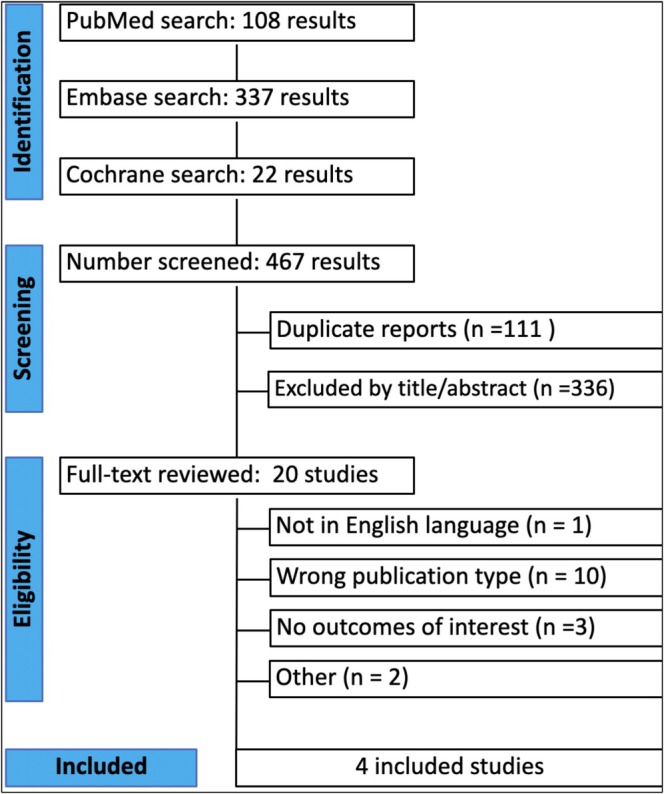
Preferred Reporting Items for Systematic Reviews and Meta‐Analyses (PRISMA) flow diagram.

Figure 1 shows the PRISMA flow diagram outlining the study selection process. The search strategy identified 467 results. After deduplication and exclusion based on title and abstract, 29 studies remained for full‐text review. Of these, four studies met all the inclusion criteria for the analysis [15, 16, 17, 18].

### 3.2 Quality Assessment

The risk of bias was assessed using the ROBINS‐I tool for observational studies and the RoB 2.0 tool for RCTs (Figure S1). Among the observational studies, both Li et al. [16] and Wei et al. [17] were judged to have an overall moderate risk of bias, primarily due to concerns related to confounding (D1) and participant selection (D2). For all other domains (D3 to D7), these studies were rated as having a low risk of bias.

In contrast, the two randomized controlled trials [15, 18] demonstrated low risk of bias across all evaluated domains, including randomization, deviations from the intended intervention, missing outcome data, measurement of outcomes, and selection of reported results. These findings suggest a generally high methodological quality among the included RCTs and acceptable quality among the observational studies, supporting the overall robustness of the meta‐analysis results.

### 3.3 Patient Characteristics

The baseline characteristics of individual studies are presented in Table 1. Two observational and two RCTs were included in this meta‐analysis, encompassing 483 patients. Of these, 249 patients received MWA combined with chemotherapy and 234 received chemotherapy alone. The number of patients in each study ranged from 49 to 293. Patient age ranged from 59 to 65 years. Most patients had advanced pulmonary adenocarcinoma.

**TABLE 1:** Baseline characteristics of included studies.

Study	Country	Study design	Sample size, M + C/C, *n*	Male M + C/C, *n*	Age M + C/C, median or mean	Adenocarcinoma M + C/C, *n*	Tumor size M + C/C, cm, mean	ECOG 0–1 M + C/C	Stage IV M + C/C, *n*	Smoker M + C/C, *n*	Follow up (months)
Shan 2021 [15]	China	RCT	34/33	24/22	72/72	14/15	3.8/3.9	NR	34/33	NR	6
Li 2019 [16]	China	Observational	21/28	10/12	60/60	21/28	3.5/4.7	20/25	21/28	5/9	36
Wei 2015 [17]	China	Observational	46/48	27/38	59/59	36/24	3.7/4.3	43/26	38/22	21/18	36
Wei 2020 [18]	China	RCT	148/145	96/93	59/61	116/107	3.6/3.7	146/141	117/113	91/79	13

Abbreviations: ECOG, Eastern Cooperative Oncology Group performance status; M + C/C, microwave ablation combined with chemotherapy/chemotherapy alone; NR, not reported; RCT, randomized controlled trial.

### 3.4 Primary Outcome

**FIGURE 2:**
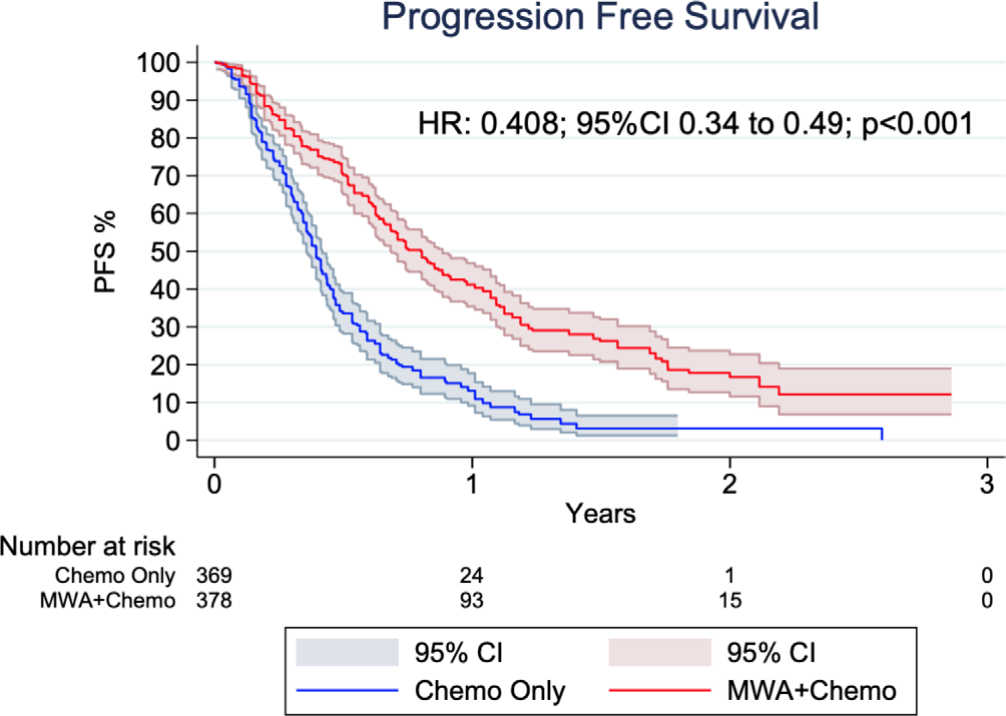
Kaplan–Meier curves showing Progression‐Free Survival (PFS) in patients treated with microwave ablation (MWA) combined with chemotherapy versus chemotherapy alone.

Table 2 summarizes the meta‐analysis findings. Figure 2 highlights a significant improvement in PFS among patients receiving MWA combined with chemotherapy compared to chemotherapy alone (HR 0.408; 95% CI 0.24–0.49; *p* < 0.001; *I* ^2^ = 53.3%; Figure 2 and Table 2). The robustness of the results was confirmed through a two‐stage meta‐analysis and leave‐one‐out sensitivity analysis, both showing consistent benefit regardless of study exclusion (Figures S2 and S3). No significant asymmetry was observed in the funnel plot (Figure S4).

**TABLE 2:** Summary of outcomes.

Outcome	Number of studies	Number of patients	Effect estimate, random model (95% CI, *p*)
Progression‐free survival	4	483	HR 0.408; 95% CI 0.24–0.49; *p* < 0.001, *I* ^2^ = 53.3%
Partial remission	3	195	RR 0.74; 95% CI 0.37–1.50; *p* = 0.408, *I* ^2^ = 62.8%
Adverse effects	4	483	RR 1.08; 95% CI 0.86–1.36; *p* = 0.491, *I* ^2^ = 56.8%

Abbreviations: CI, confidence interval; HR, hazard ratio; RR, relative risk.

### 3.5 Secondary Outcomes

**FIGURE 3:**
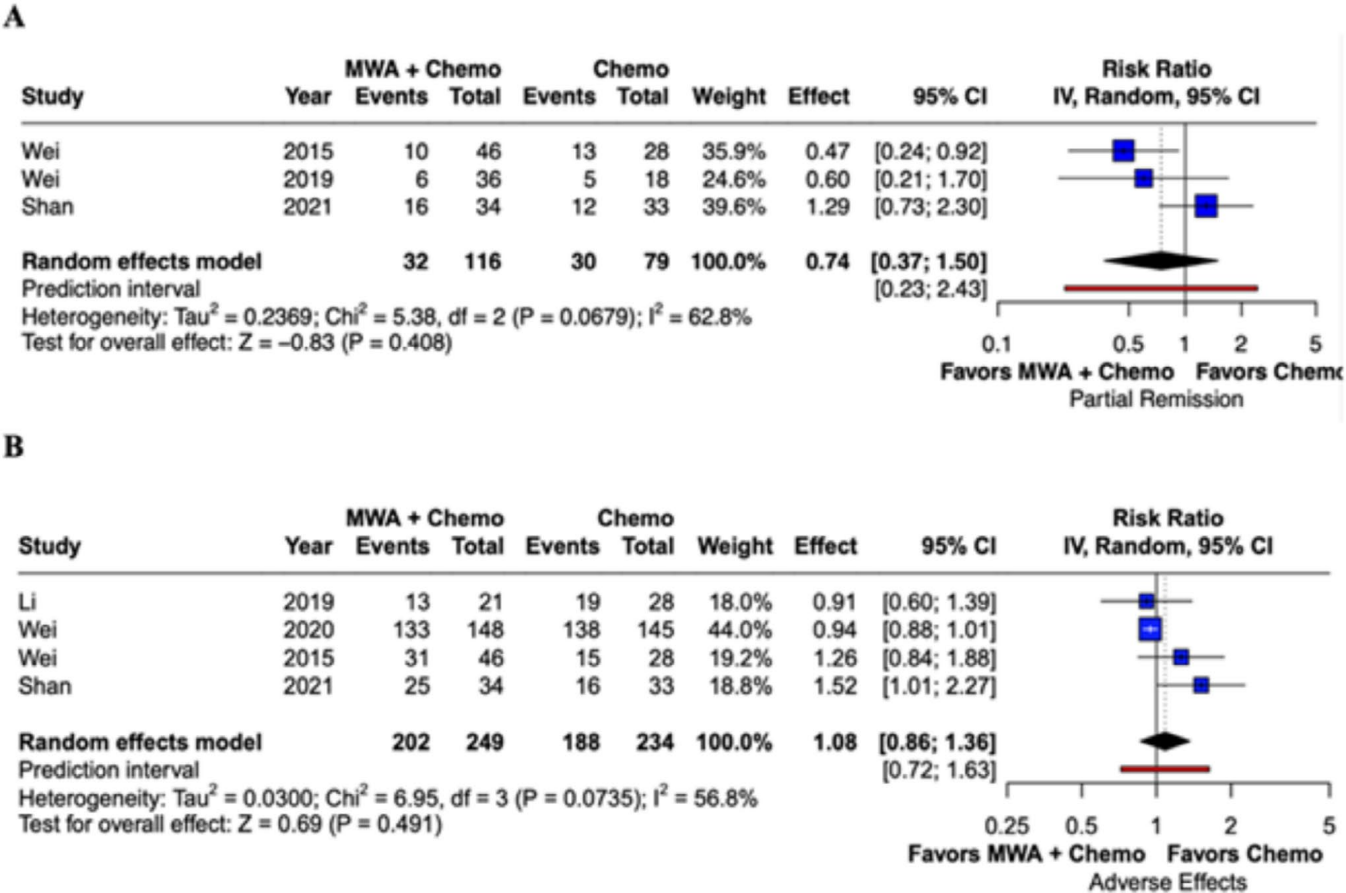
Forest plots comparing microwave ablation (MWA) combined with chemotherapy versus chemotherapy alone. (A) Partial Remission; (B) Adverse Effects.

The partial remission rate did not differ significantly between groups, as showed in Figure 3A (RR 0.74; 95% CI 0.37–1.50; *p* = 0.41; *I* ^2^ = 62.8%; Figure 3A and Table 2).

The incidence of adverse events was comparable between groups, as demonstrated in Figure 3B (RR 1.08; 95% CI 0.86–1.36; *p* = 0.49; *I* ^2^ = 56.8%; Figure 3B and Table 2).

## 4 Discussion

In this systematic review and meta‐analysis of four studies including 483 patients, we comprehensively analyzed the outcomes of MWA combined with chemotherapy compared to chemotherapy alone in patients with advanced NSCLC. Our main findings were as follows: (I) patients receiving MWA combined with chemotherapy demonstrated a significant improvement in PFS; (II) there was no significant difference in the partial remission rate between groups; and (III) there was no significant difference in the rate of adverse events. Importantly, definitions and assessment criteria for partial remission rate and adverse events varied across the included studies, which may have introduced heterogeneity and influenced the pooled effect estimates for these secondary outcomes.

NSCLC is known to develop through progressive pathological changes and presents with distinct molecular features, including characteristic genomic alterations. These changes typically originate in the epithelial cells lining the airways, which are constantly exposed to carcinogens and environmental pollutants such as asbestos. The tumorigenic process evolves through several precancerous stages, including hyperplasia, squamous metaplasia, squamous dysplasia, and eventually carcinoma in situ, before progressing to invasive cancer [5]. These complexities, along with the high burden of advanced‐stage disease, motivated our meta‐analysis, which pooled data from both randomized and non‐randomized studies to evaluate whether MWA, when combined with chemotherapy, could improve survival outcomes in patients with advanced NSCLC compared to chemotherapy alone.

Among the studies included, Shan et al. [15] demonstrated that performing MWA during chemotherapy was more effective in improving PFS than delivering MWA after chemotherapy. This early reduction in tumor burden appears to contribute to improved tumor‐free survival and enhanced quality of life, including mental well‐being. Our meta‐analysis supports this finding, showing a statistically significant benefit in PFS with the combination of MWA and chemotherapy. Similarly, Xu et al. [2] reported that although overall survival did not differ significantly between the treatment groups, a substantial improvement in PFS was observed, aligning with our findings.

Li et al. [16] also demonstrated the benefits of combining MWA with chemotherapy in stage IV lung adenocarcinoma, reporting improved PFS and time to local progression (TTLP). Our results are consistent regarding PFS, though we did not find significant data supporting the TTLP benefit. Furthermore, Wei et al. [17] reported improved PFS in patients with advanced NSCLC treated with MWA combined with chemotherapy, reinforcing the evidence from our pooled analysis. This trend was further confirmed by Wei et al. [18], who observed longer PFS and OS in patients receiving the combined treatment, compared to chemotherapy alone.

In our systematic review, adenocarcinoma was the most common histological subtype, more prevalent than squamous cell carcinoma, which is consistent with the distribution reported by Wei et al. [18], where 116 out of 293 patients had adenocarcinoma. MWA combined with chemotherapy appears to be a promising therapeutic option, especially in patients who are not candidates for surgery. The appeal of this combination lies in its potential synergy to improve PFS and possibly OS, which are critical endpoints in the management of advanced NSCLC. These results suggest that this approach is safe, although it did not significantly impact other outcomes such as disease control rate (DCR), adverse events, or partial remission.

Additionally, Kakamad et al. [19] suggested that MWA combined with chemotherapy may offer a more effective strategy to reduce recurrence and limit complications, with a treatment response profile comparable to chemotherapy alone. Nonetheless, despite its theoretical advantages and encouraging results for PFS, clinical outcomes of MWA combined with chemotherapy in advanced NSCLC have shown variability across studies. This inconsistency may be attributed to differences in patient selection, MWA protocols, chemotherapy regimens, and operator experience across institutions. Furthermore, the biological mechanisms underlying the potential synergistic effect of MWA and chemotherapy remain to be fully elucidated, representing a fertile area for ongoing investigation.

### 4.1 Study Strength and Limitations

This is the first meta‐analysis specifically focused on evaluating MWA combined with chemotherapy in patients with advanced NSCLC. Its primary strength lies in the comprehensive inclusion of both randomized and non‐randomized studies, enabling a broader assessment of the available evidence. However, this work is subject to the intrinsic limitations of a study‐level meta‐analysis, including methodological heterogeneity and the potential for residual confounding. The lack of individual patient data prevented subgroup analyses that could have provided deeper insights. Additionally, there was notable heterogeneity in the definitions and measurements of outcomes across studies. The observed heterogeneity was moderate and may be partially explained by differences in study design, timing of MWA relative to chemotherapy, and variations in tumor stage distribution across the included studies. It is also important to note that Wei et al. [18] contributed a substantially larger sample size compared to the other studies, which may have influenced the overall pooled estimates.

## 5 Conclusion

This meta‐analysis shows that MWA combined with chemotherapy significantly improves progression‐free survival in patients with advanced NSCLC, without increasing adverse events. While the results are promising, further high‐quality randomized trials are needed to confirm these findings and better define the role of MWA in this setting.

## Author Contributions

**Paul C. Onyeji:** conceptualization, data curation, formal analysis, writing – original draft. **Amrinder Kaur:** formal analysis, writing – original draft. **Leo Consoli:** data curation, investigation, writing – review and editing. **Shivank Dani:** investigation, data curation, visualization. **Sonise Momplaisir‐Onyeji:** data curation, writing – review and editing, validation. **Felipe S. Passos:** methodology, formal analysis, validation, risk of bias assessment, writing – review and editing. **Torsten Doenst:** supervision, methodology, writing – review and editing. **Hristo Kirov:** methodology, formal analysis, writing – review and editing. **Tulio Caldonazo:** project administration, supervision, writing – review and editing.

## Funding

T.C. was funded by the Deutsche Forschungsgemeinschaft (DFG, German Research Foundation) Clinician Scientist Program OrganAge funding number 413668513, by the Deutsche Herzstiftung (DHS, German Heart Foundation) funding number S/03/23 and by the Interdisciplinary Center of Clinical Research of the Medical Faculty Jena.

## Disclosure

The authors have nothing to report.

## Conflicts of Interest

The authors declare no conflicts of interest.

## Supporting information


**Table S1:** Complete search strategy.
**Figure S1:** Risk of bias assessment. (A) ROBINS‐I tool for observational studies; (B) RoB‐2 tool for RCTs.
**Figure S2:** Two‐Stage Survival Meta‐Analysis Forest Plot.
**Figure S3:** Leave‐one‐out sensitivity analysis for primary outcome.
**Figure S4:** Funnel Plot for Publication Bias Assessment.

## Data Availability

The data that supports the findings of this study are available in Supporting Information of this article.

## References

[1] J. Ferlay , M. Ervik , F. Lam , et al., Global Cancer Observatory: Cancer Today (International Agency for Research on Cancer, 2020).

[2] R. Xu , J. Chen , D. Chen , et al., “CT‐Guided Percutaneous Microwave Ablation Combined With Local Radiotherapy or Chemotherapy of Malignant Pulmonary Tumors,” Current Radiopharmaceuticals 17, no. 2 (2024): 184–199. 38204263 10.2174/0118744710261655231214105406 PMC11327768

[3] A. M. Moussa , E. Ziv , S. B. Solomon , and J. C. Camacho , “Microwave Ablation in Primary Lung Malignancies,” Seminars in Interventional Radiology 36, no. 4 (2019): 326–333, 10.1055/s-0039-1700567. 31680724 PMC6823043

[4] Y. H. Sun , P. Y. Song , Y. Guo , et al., “Effects of Microwave Ablation or Its Combination With Whole‐Body Chemotherapy on Serum Vascular Endothelial Growth Factor Levels in Patients With Stage IIIB/IV NSCLC,” Genetics and Molecular Research 14, no. 3 (2015): 10015–10025. 26345938 10.4238/2015.August.21.8

[5] Q. Guo , L. Liu , Z. Chen , et al., “Current Treatments for Non‐Small Cell Lung Cancer,” Frontiers in Oncology 12 (2022): 945102, 10.3389/fonc.2022.945102. 36033435 PMC9403713

[6] S. K. Das , Y. Y. Huang , B. Li , X. X. Yu , R. H. Xiao , and H. F. Yang , “Comparing Cryoablation and Microwave Ablation for the Treatment of Patients With Stage IIIB/IV Non‐Small Cell Lung Cancer,” Oncology Letters 19, no. 1 (2020): 1031–1041. 31885721 10.3892/ol.2019.11149 PMC6924207

[7] K. Feng and Y. Lu , “Clinical Analysis of Systemic Chemotherapy Combined With Microwave Ablation in the Treatment of Lung Cancer,” Asian Journal of Surgery 45, no. 5 (2022): 1107–1112. 34509354 10.1016/j.asjsur.2021.08.013

[8] K. B. Gala , N. S. Shetty , P. Patel , and S. S. Kulkarni , “Microwave Ablation: How We Do it?,” Indian Journal of Radiology and Imaging 30, no. 2 (2020): 206–213, 10.4103/ijri.IJRI_240_19. 33100690 PMC7546284

[9] M. J. Page , J. E. McKenzie , P. M. Bossuyt , et al., “The PRISMA 2020 Statement: An Updated Guideline for Reporting Systematic Reviews,” BMJ 372 (2021): n71. 33782057 10.1136/bmj.n71 PMC8005924

[10] M. J. Page , D. Moher , P. M. Bossuyt , et al., “PRISMA 2020 Explanation and Elaboration: Updated Guidance and Exemplars for Reporting Systematic Reviews,” BMJ 29 (2021): n160. 10.1136/bmj.n160 PMC8005925 33781993

[11] J. A. Sterne , M. A. Hernán , B. C. Reeves , et al., “ROBINS‐I: A Tool for Assessing Risk of Bias in Non‐Randomised Studies of Interventions,” BMJ 355 (2016): i4919. 27733354 10.1136/bmj.i4919 PMC5062054

[12] J. A. C. Sterne , J. Savović , M. J. Page , et al., “RoB 2: A Revised Tool for Assessing Risk of Bias in Randomised Trials,” BMJ 366 (2019): l4898. 31462531 10.1136/bmj.l4898

[13] Y. Wei and P. Royston , “Reconstructing Time‐to‐Event Data From Published Kaplan–Meier Curves,” Stata Journal: Promoting Communications on Statistics and Stata 17, no. 4 (2017): 786–802. PMC5796634 29398980

[14] M. K. Goel , P. Khanna , and J. Kishore , “Understanding Survival Analysis: Kaplan–Meier Estimate,” International Journal of Ayurveda Research 1, no. 4 (2010): 274. 21455458 10.4103/0974-7788.76794 PMC3059453

[15] Y. Shan , X. Yin , F. Lin , C. Wang , Y. Kong , and W. Yao , “Chemotherapy Combined With Intermittent Microwave Ablation in the Treatment of Oligometastatic Non‐Small Cell Lung Cancer,” Journal of Balkan Union of Oncology 26, no. 2 (2021): 320–327. 34076975

[16] C. Li , J. Wang , J. B. Shao , L. M. Zhu , Z. G. Sun , and N. Zhang , “Microwave Ablation Combined With Chemotherapy Improved Progression Free Survival of IV Stage Lung Adenocarcinoma Patients Compared With Chemotherapy Alone,” Thoracic Cancer 10, no. 7 (2019): 1628–1635. 31243894 10.1111/1759-7714.13129 PMC6610256

[17] Z. Wei , X. Ye , X. Yang , et al., “Microwave Ablation Plus Chemotherapy Improved Progression‐Free Survival of Advanced Non‐Small Cell Lung Cancer Compared to Chemotherapy Alone,” Medical Oncology 32 (2015): 1–8. 10.1007/s12032-014-0464-z 25572816

[18] Z. Wei , X. Yang , X. Ye , et al., “Microwave Ablation Plus Chemotherapy Versus Chemotherapy in Advanced Non‐Small Cell Lung Cancer: A Multicenter, Randomized, Controlled, Phase III Clinical Trial,” European Radiology 30 (2020): 2692–2702. 32020400 10.1007/s00330-019-06613-x

[19] F. H. Kakamad , R. M. Ali , S. H. Tahir , et al., “Microwave Ablation With or Without Chemotherapy in Management of Non‐Small Cell Lung Cancer: A Systematic Review,” Barw Medical Journal 3, no. 1 (2024).

