# Supplementary material for: Microwave Ablation Combined With Chemotherapy Versus Chemotherapy Alone in Patients With Advanced Non‐Small Cell Lung Cancer—Systematic Review and Meta‐Analysis
Source: Thorac Cancer. 2026 Jan 9;17(1):e70221. doi: 10.1111/1759-7714.70221 (PMC12789646; doi:10.1111/1759-7714.70221)
Supplement: Supplementary file 1 — Table S1: Complete search strategy. Figure S1: Risk of bias assessment. (A) ROBINS‐I tool for observational studies; (B) RoB‐2 tool for RCTs. Figure S2: Two‐Stage Survival Meta‐Analysis Forest Plot. Figure S3: Leave‐one‐out sensitivity analysis for primary outcome. Figure S4: Funnel Plot for Publication Bias Assessment. [file TCA-17-e70221-s001.docx]

**Supplementary material**

**Supplementary Table 1.** Complete search strategy

**Supplementary Figure 1.** Risk of bias assessment. A) ROBINS-I tool for observational studies; B) RoB-2 tool for RCTs.

**Supplementary Figure 2.** Two-Stage Survival Meta-Analysis Forest Plot

**Supplementary Figure 3.** Leave-one-out sensitivity analysis for primary outcome

**Supplementary Figure 4.** Funnel Plot for Publication Bias Assessment

**Supplementary Table 1**

(non-small cell lung cancer OR NSCLC OR lung adenocarcinoma) AND (microwave ablation OR MWA OR thermal ablation) AND (immunotherapy OR immune checkpoints inhibitors OR chemotherapy OR antineoplastic agents OR systemic therapy)


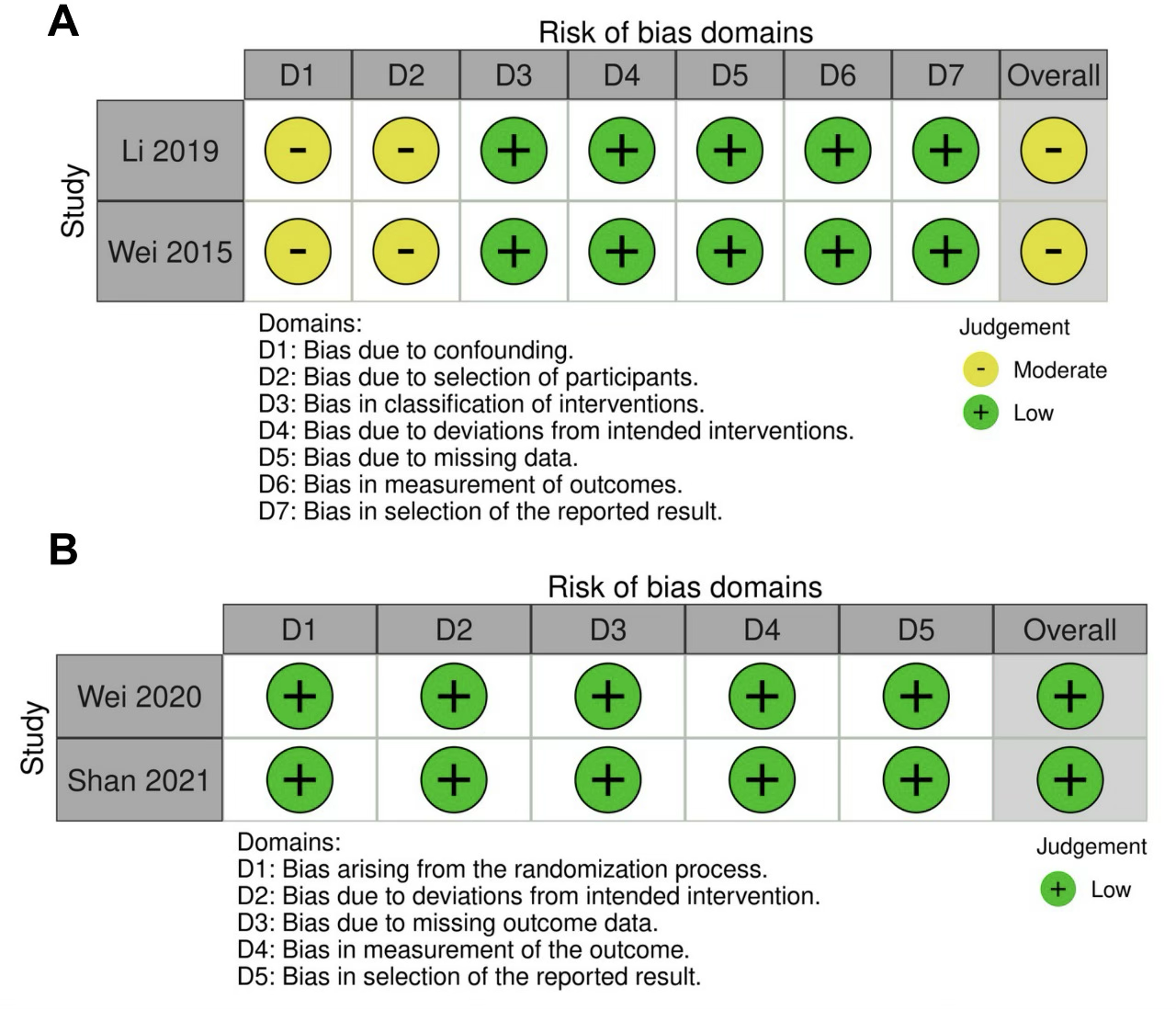
**Supplementary Figure 1**

**Supplementary Figure 2**


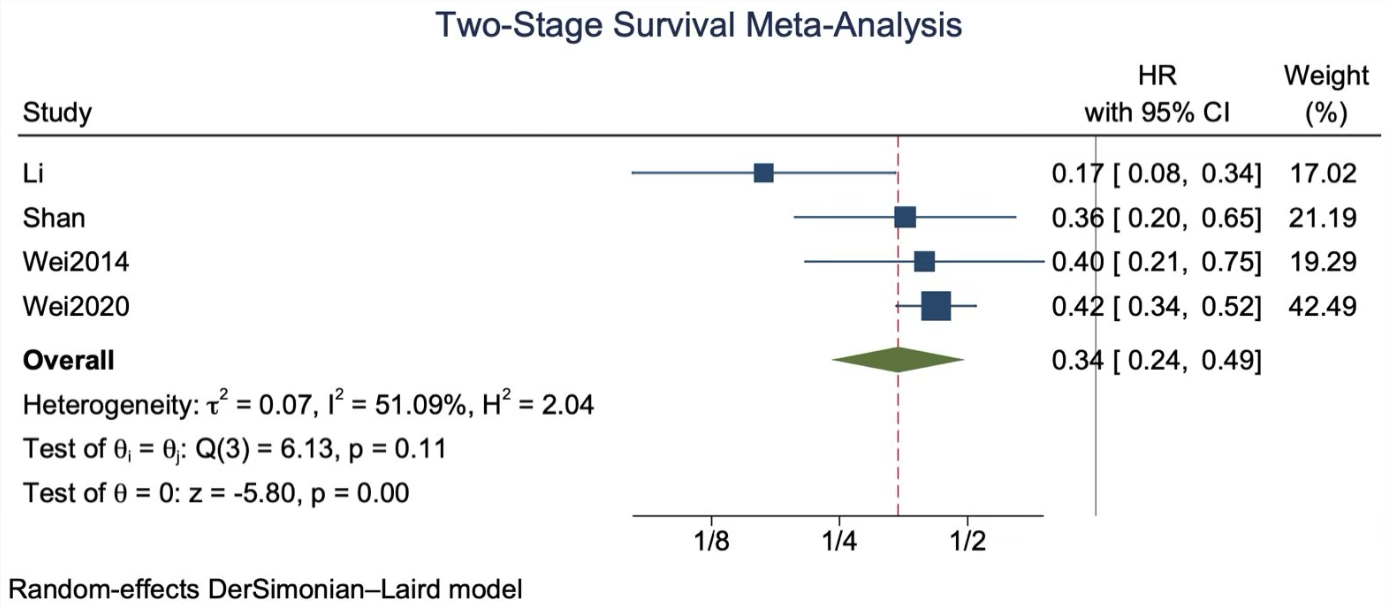


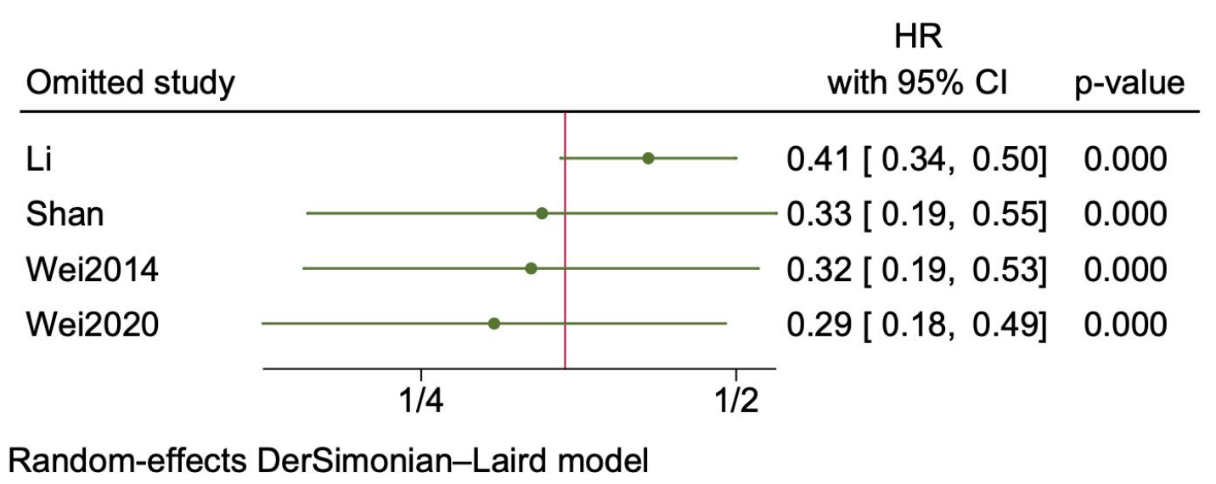
**Supplementary Figure 3**


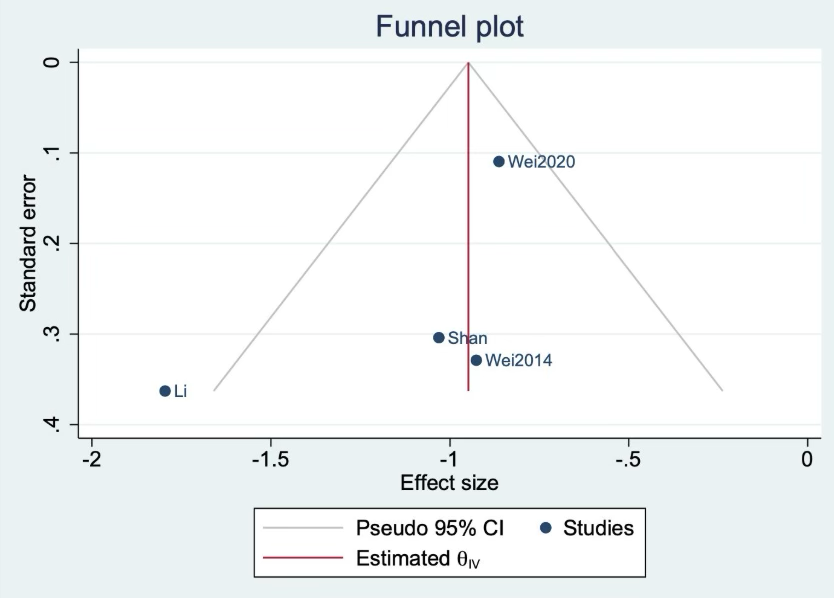
**Supplementary Figure 4**
